# The characteristics of HIV-1 subtype B on phylogenetic dynamic and molecular transmission network in Fuyang City, China, 2011 to 2019

**DOI:** 10.3389/fpubh.2023.1092376

**Published:** 2023-03-01

**Authors:** Wenting Pan, Nannan Gao, Bing Hu, Yueqi Yin, Yuelan Shen, Xiaohui Yang, Wei Wei, Jie Ni, Seying Dai, Lifeng Miao, Yizu Qin, Lin Jin, Hongxiong Guo, Jianjun Wu

**Affiliations:** ^1^Anhui No. 2 Provincial People's Hospital, Hefei, China; ^2^Department of Health Inspection and Quarantine, School of Public Health, Anhui Medical University, Hefei, China; ^3^Department of AIDS Prevention and Control, Fuyang Center for Disease Control and Prevention, Fuyang, China; ^4^School of Medicine, Ningbo University, Ningbo, China; ^5^Department of AIDS Prevention and Control, Anhui Provincial Center for Disease Control and Prevention, Hefei, China; ^6^NHC Key Laboratory of Enteric Pathogenic Microbiology, Jiangsu Provincial Center for Disease Control and Prevention, Nanjing, China

**Keywords:** phylodynamic, molecular transmission network, HIV-1, subtype B, Fuyang

## Abstract

**Introduction:**

HIV-1 subtype B, as once one of the earliest strains introduced into mainland China rapidly spread in commercial plasma donors and heterosexuals in 1990s. Here, we aim to investigate the origin and evolutionary history of HIV-1 subtype B in Fuyang city, China.

**Methods:**

We collected sequences tested from Fuyang in the east of China where higher prevalence of HIV-1 among commercial plasma donors and heterosexuals to construct a phylogenetic tree using the Markov chain Monte Carlo (MCMC) algorithm, infer molecular transmission network using TN93 model and visualize it with Cytoscape software.

**Results and discussion:**

Our results showed that >99% of subtype B sequences belonged to Thai B. The sequences from Fuyang often cluster closer to those from other its adjacent cities, which clustered together and formed a monophyletic cluster. HIV-1 B circulating in Fuyang dates back to approximately 1990. Among the 1,437 sequences, 166 clustered at a genetic distance of ≤1.2%, resulting in 73 clusters. The degree of clustering with at least one other person was 11.55%. Among the transmission clusters, 50 (80.65%) comprised two individuals. Most clusters consisted of both heterosexual transmission routes and men who have sex with men. Phylogenetic and molecular network analyses revealed a common origin with neighboring regions in mainland China, local onwards transmission after its introduction, and a limited clustering degree. However, at least two co-existing transmission routes in most transmission clusters imply a greater challenge in controlling the spread of HIV-1. Our findings highlight the value on tailoring prevention interventions by combination of molecular surveillance and epidemiology.

## 1. Introduction

According to the Global acquired immune deficiency syndrome (AIDS) update 2022 released by UNIAIDS (https://www.unaids.org/sites/default/files/media_asset/2022-global-aids-update_en.pdf), it shows that 1.5 million people were newly diagnosed with HIV infection and 650,000 people died of AIDS-related diseases in 2018. HIV-1 is genetically characterized by its extensive diversification and rapid evolution. The current HIV-1 pandemic is phylogenetically divided into four groups: M, N, O, and P. M groups are predominant and are divided into nine subtypes (A, B, C, D, F, G, H, J, and K) and 118 circulating recombinant forms (CRFs) (https://www.hiv.lanl.gov/content/sequence/HIV/CRFs/CRFs.html). During 2000–2007, HIV-1 subtype B accounted for 11–12% of all M group HIV-1, and it prevailed mainly in Australia, Western Europe, Latin America, the Caribbean Sea region, Central Europe, and North America (1, 2). The HIV-1 subtype B strain was first reported in China in the 1980s (3, 4). They are divided into four subgroups: Thai-B (B'), pandemic B (Pan B), Beijing-B (BJ-B), and Taiwan-B (TW-B). HIV-1 B' is the most widespread HIV-1 strain in mainland China in terms of geographic reach and has been identified in 28 provinces (3). Although it belongs to subtype B, it is separated on the phylogenetic tree from HIV-1 subtype B circulating in the Occident (5). In mainland China, Thai-B, which was identified among intravenous drug users living in Yunnan province in China in 1989 (4), was introduced into central China and became a major source of HIV-1 spreading among plasma donors (5, 6).

The first HIV-1 molecular surveillance study was conducted in China between 1996 and 1998. The results showed that Thai B accounted for 47.16%, and Pan-B accounted for only 5.67% (7). The second HIV-1 molecular surveillance conducted in 2007–2008 showed that HIV-1 B' declined to 35.86% (8), and the other HIV-1 B strains reduced to 1.66%. HIV-1 B' decreased further to 17.44%, while the other HIV-1 B strains increased to 3.91%. However, HIV-1 B' was predominant among plasma donors (92.5 %). It had spread to 28 of 34 provinces in China, especially in central and southwest China, where HIV-1 B' was the main virus circulating in the population with HIV infection *via* a heterosexual transmission route. Nonetheless, there is little data to report molecular phylogenetic dynamic and molecular transmission network of subtype B in China.

Fuyang City is located in the east of China, affiliated to Anhui Province. HIV-1 was first identified among commercial blood donors and the heterosexual transmission population in the 1990s as well as in other developing cities in China (9). As of 31 December 2020, a total of 4,733 individuals living with HIV/AIDS have been reported. Of them, 3,186 developed AIDS. Of all HIV subtypes, HIV-1 B accounts for only 30% of all HIV-1 strains circulating in Fuyang. As the earliest HIV-1 emerged and prevailed in Fuyang, the studies focusing HIV-1 B' phylogenetic history and transmission trajectory was little as well as in other places of China, it is obvious distinct with CRF01_AE and CRF07_BC (10–12). Here, we described the features of phylogenetic dynamic and molecular transmission of the HIV-1 B in near 10 years.

## 2. Materials and methods

### 2.1. The subjects, sequence data and subtype identification

All subjects were diagnosed as HIV-1 positive in 2005 and 2011–2019, were registered permanent residents, and lived in Fuyang City. Drug resistance surveillance is regularly conducted among HIV-1 infected people with antiviral therapy. The segment of pol gene was sequenced according to previous protocol (10, 13). If there was more than one sequence for the same person, only the earliest sequence was selected for phylogenetic and molecular transmission network analyses. All sequences with lengths >987bp were used.

### 2.2. The construction of the phylogenetic tree

Nucleotide sequences were aligned with reference strains using the Clustal W program implemented in the MEGA11.0 software. Reference strains were downloaded from GenBank, which includes the sequences of HIV-1 subtype B circulating worldwide during 1983–1995 and those having the highest similarity with Fuyang sequences identified during 1983–2019. We used MEGA11.0 software to calculate the gene distance of Fuyang sequences. Because the distance between sequences was < 2%, only two of them were used for phylogenetic analysis. To assess the appropriate model of evolution for the phylogenetic analysis of pol gene datasets, likelihood ratio tests were conducted using the jModel Test program based on the Akaike information criterion (AIC). Both the general time-reversal (GTR) substitution with a gamma distribution model of site rate heterogeneity were employed to conduct Bayesian coalescent analyses using the Markov chain Monte Carlo (MCMC) algorithm with BEAST software (version 1.82). The MCMC search was run for 5 × 10^6^ generations with trees sampled every 100^th^ generation. Burn-in was set at 20%, and a posterior consensus tree was generated from 50,000 sampled trees. The estimated effective sampling sizes (ESSs) were evaluated to assess the sampling convergence of MCMC procedures. The Tracer program (version 1.5) was used to interpret the MCMC chains and output posterior trees.

### 2.3. Network analysis

**Figure 1:**
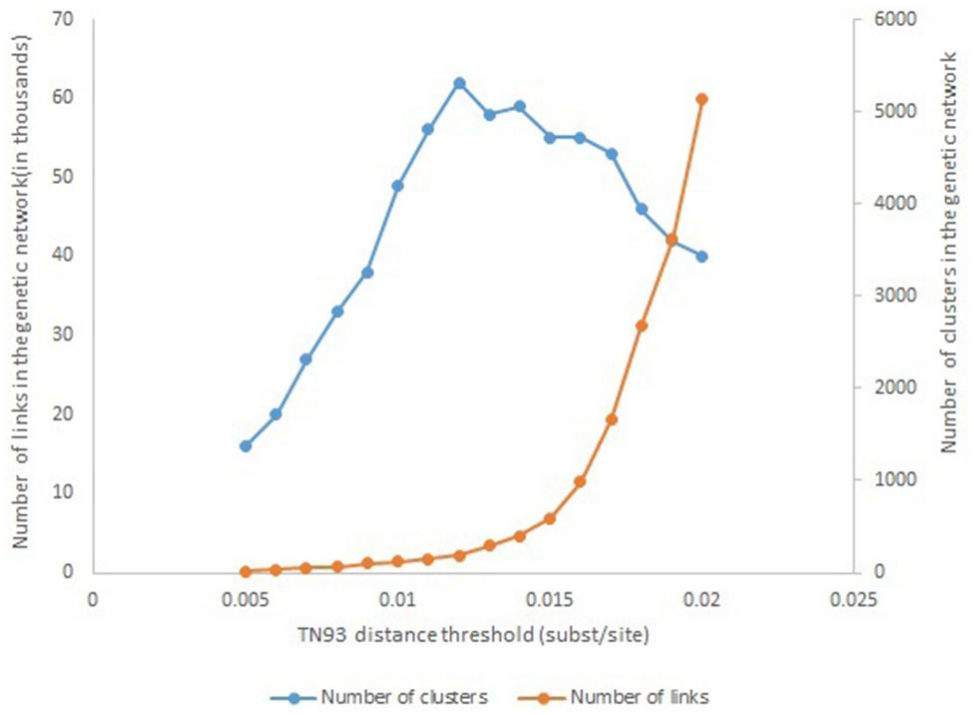
Number of genetic links and transmission clusters, as a function of the TN93 distance threshold.

To infer the molecular network and genetic distance, the Tamura-Nei 93 nucleotide substitution model (TN93), was used to calculate pairwise distance between all pairs of HIV pol sequences using HYPHY version 2.2.4. The number of 1.2% substitutions/site was used as a threshold to construct the genetic network because this distance identifies the maximum number of clusters in the genetic network (Figure 1). The network data were visualized using Cytoscape software version 3.8.

## 3. Results

### 3.1. The population characteristics

A total of 1,437 subjects were included in the study. Of these, 60.32% were male. Heterosexuality was the predominant transmission route, followed by men who have sex with men (MSM). Of these, 35.70% developed into AIDS and 44.62 and 24.35% of these patients received middle school and high school education, respectively. Only 13.35% of them had received primary school education (Table 1).

**Table 1:** The basic characteristics of population with HIV-1 living in Fuyang, China.

**Variable**	**Number**	**Percent (%)**
**Age**		
20–39	375	26.13
40–49	407	28.36
50–69	504	35.08
70–	149	10.43
**Sex**		
Male	867	60.32
Female	570	39.68
**Transmission route**		
Homosexual	558	38.83
Heterosexual	856	59.57
Blood	16	1.11
Unknown	7	0.49
**Current status**		
HIV-1 infectors	924	64.30
Patients with AIDS	513	35.70
**Education level**		
Primary school	192	13.35
Middle school	641	44.62
High school	350	24.35
Undergraduate and over	254	17.68

### 3.2. Phylogenetic analysis

**Figure 2:**
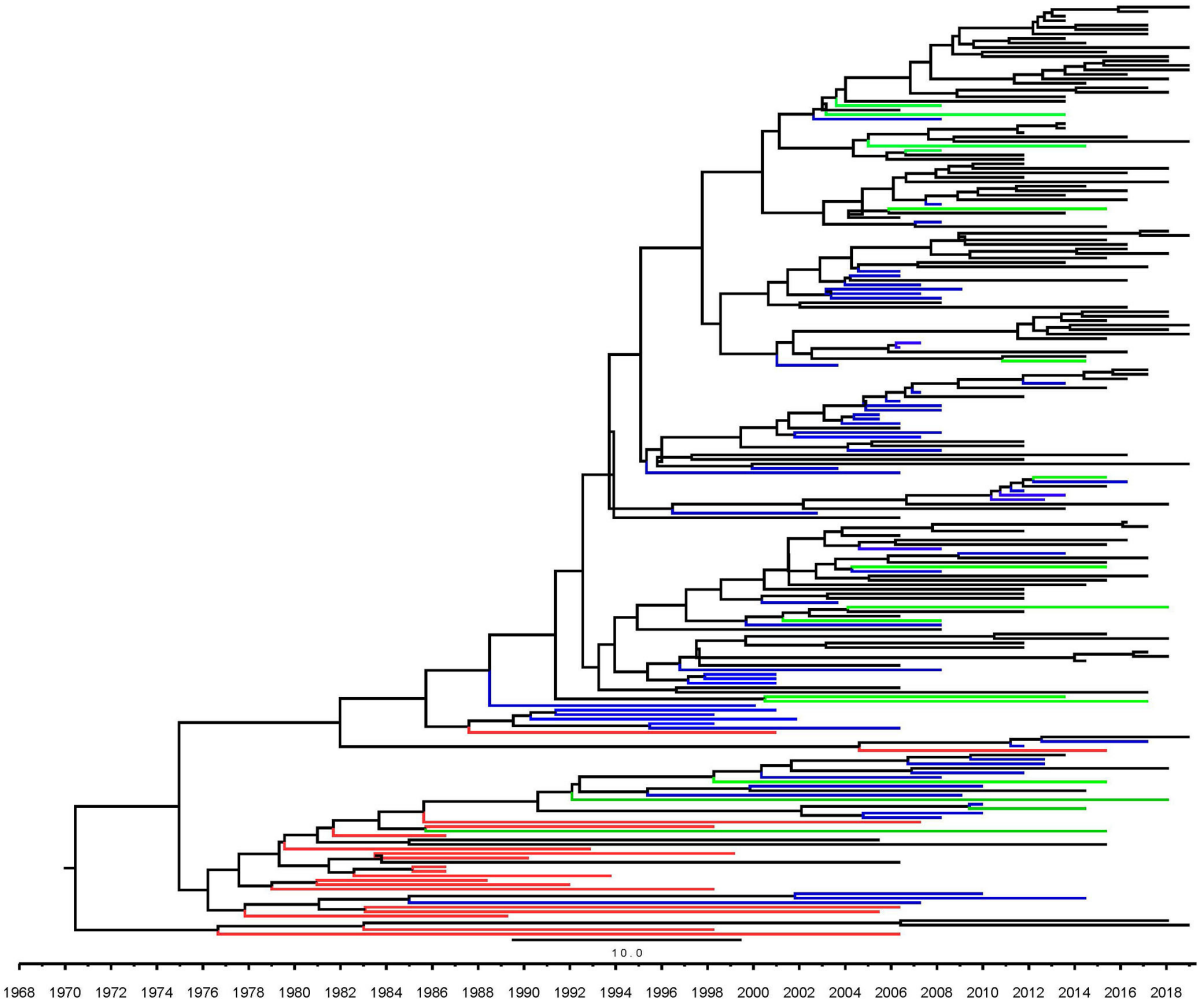
Maximum clade credibility trees based on partial *pol* gene segment of HIV-1. Black line indicates the sequence of HIV-1 from Fuyang city, green line indicates the sequences from other cities in Anhui province, blue line indicates the sequences isolated from other provinces of China except Anhui, red line indicates sequences from other countries.

Among the HIV-1 subtype B strains circulating in Fuyang, which was divided into two clusters. More than 99% of them belonged to HIV-1 B' which was designated as cluster 1 (Figure 2), with the rest being Pan-B (cluster 2). Most of the sequences clustered together to form a monophyletic cluster. The sequences isolated from Fuyang often clustered closer to those from other cities located in Anhui, Henan, and Hebei provinces than to those from other provinces in China. Five sequences isolated in 2011, 2013, 2016, and 2019 had longer branches in the phylogenetic tree. HIV-1 B' circulating in Fuyang dates back to about 1990 (Figure 2).

### 3.3. Network analysis

**Figure 3:**
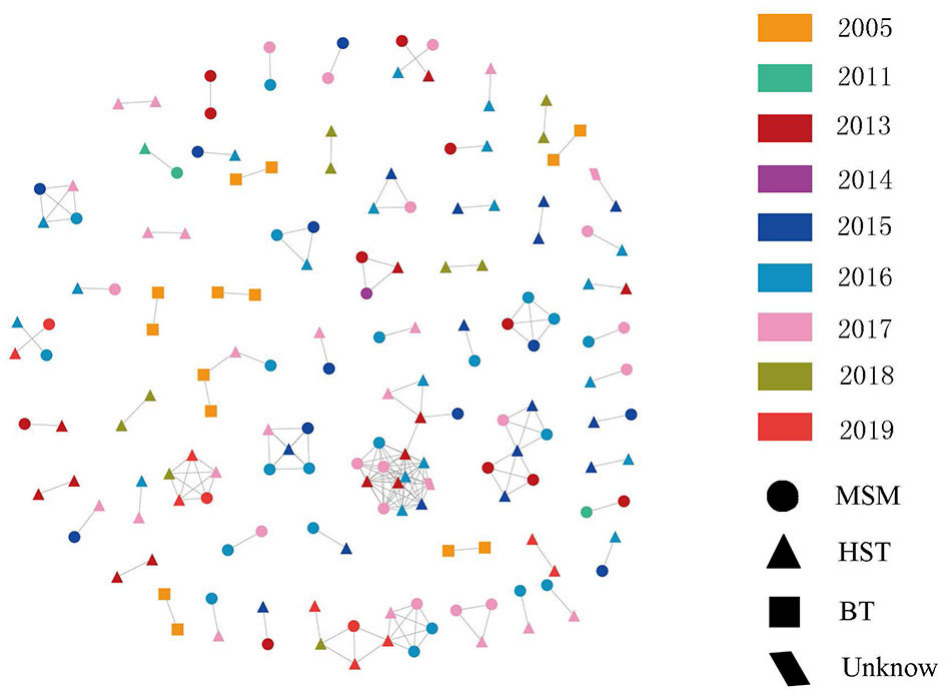
Fuyang City HIV-1 surveillance molecular transmission network. Links (i.e., edges) indicate genetic distance ≤ 0.015 substitutions/site. Color indicates diagnosed time. Shape indicated transmission route. HST, heterosexual; MSM, men who have sex with men; BT, blood transmission route.

Among the 1,437 sequences, 166 fell into clusters at a genetic distance of ≤1.5%, resulting in 73 clusters ranging in size from two to five individuals (Figure 3). The degree of clustering (clustered with at least one other person) was 11.55% (166/1,437). Among the transmission clusters, 50 (80.65 %) comprised two individuals. Among all clusters, blood transmission is involved in seven networks. Of these, six networks were formed by two individuals who received HIV-1 infection *via* blood transmission, and one cluster was formed by four individuals consisting of heterosexuals and MSM. Most clusters consisted of both heterosexual transmission routes and MSM. Among the 55 networks formed by heterosexual transmission route and/or MSM, only six were formed by only heterosexual transmission route or MSM, and the rest included both heterosexual transmission routes and MSM. Among all sequences in the transmission networks, 39.16% were isolated from MSM and 51.20% were isolated from heterosexuals (Figure 3). This was close to the proportion of MSM and heterosexual transmission routes among all the subjects in this study. Among the 73 clusters, the time depth ranged 1–4 years. Additionally, the time depth of 39.73% of the clusters was within 1 year and the median time depth was 2 years. The age span of the transmission clusters ranged 0–48 years, with an average of 13.56 years.

## 4. Discussion

Four types of HIV-1 subtype B were introduced in China in the 1980s (3). Nonetheless, it is unclear which of the HIV-1 B clades was seeded earlier in China. HIV-1 B' has gradually become the predominant HIV-1 strain circulating among blood donors and a heterosexual risk population in the early phase of HIV prevalence in China (4). In this study, we found that >99% of HIV-1 subtype B circulating in Fuyang belongs to B'. They were dated to approximately 1990 (Figure 2). Most sequences isolated from Fuyang formed a few monophyletic clusters. It was revealed that HIV-1 B' circulating in Fuyang was mainly local onward transmission after it was introduced as well as HIV-1 spread in most cities previously reported (14–16). Moreover, the sequences of HIV-1 B' circulating in Fuyang are close to those of strains isolated from the center of China, such as Henan and Hubei, where the earliest HIV-1 was found in commercial blood donors in the early phase of HIV-1 prevalence, and B' was the predominant clades (9, 17). This suggests that they may have been epidemically linked during the early phase of HIV-1 transmission. Fuyang City borders Henan Province in the west, southwest, and northwest, and Henan Province was the second-highest HIV-1 prevalent region in the early HIV spread phase in China, where commercial blood donors have been the major high-risk population for HIV-1 infection. Furthermore, the sequences from these regions clustered together in the phylogenetic tree (Figure 2). This indicates that HIV-1 introduction into these regions may share common sources and/or routes.

Among people with HIV-1 subtype B living in Fuyang City, the heterosexual transmission route was predominant, and most of them received an education lower than high school. This was different from those in some developed regions in China, where the proportion of MSM and heterosexual route was similar in the HIV-infected population (18). Although intravenous drug users (IDUs) are one of the major high-risk populations infected with HIV-1, HIV-1 B' is rare among them. In this study, we found no circulating HIV-1 B among the IDUs. This indicates that the introduction and transmission routes of various subtypes and CRFs of HIV-1 were different. For example, CRF07_BC and CRF08_BC predominate among IDUs, whereas subtype B is predominant among blood donors and heterosexuals in the early phase of HIV-1 spread in China (19–21).

Our results showed that only 11.55% of the sequences clustered in the transmission network. 80.65% of clusters were formed by two individuals. The largest cluster size was no more than five individuals. Molecular transmission networks in Fuyang were characterized by a lower degree of clustering, smaller cluster size, and shorter time depth than those in previous studies. These features are in accordance with the persistently decreasing prevalence of HIV-1 B' strains in recent decades in China. Unlike the major HIV-1 CRFs circulating in China, the clustering degree of CRF08_BC and CRF01_AE reached 34.9% in the studies by Li et al. respectively (22, 23). In a study by Jeannette et al., the degree of clustering of subtype B across the USA was 24% (17). It is known that subtype B is predominant in the USA. Moreover, the shorter time depth here supports the notion that high HIV-1 transmissibility always occurs during the early phase of infection and before receiving antiviral treatment (24). The characteristics of the HIV-1 molecular transmission network together with its phylogenetic can not only reflect viral prevalence status and trends, but can also be used to assess the effectiveness of prevention measures (25, 26).

This study had some limitations. Sequences of HIV-1 infectors diagnosed during 2006–2010 were unavailable. Therefore, we are unable to present the molecular features of HIV-1 strains circulating in 2006–2010 and elucidate the relationship between the phylogenetic and transmission networks of HIV-1 strains in various phases. For example, some long branches were independently exposed and did not cluster with the local sequences in the phylogenetic tree (Figure 2).

## 5. Conclusion

To our knowledge, this is the first molecular transmission network analysis of the HIV-1 subtype B circulating in China. Although all sequences were from Fuyang City, the characteristics presented here may be a snapshot of the long-term prevalence of HIV-1 subtype B on phylogenetic and transmission networks throughout the country.

## Data availability statement

The datasets presented in this study can be found in online repositories. The names of the repository/repositories and accession number(s) can be found in the article/supplementary material.

## Ethics statement

The studies involving human participants were reviewed and approved by the Medical Ethics Committee of Fuyang Center for Disease Control and Prevention (2020-CDC(AH)-18). The patients/participants provided their written informed consent to participate in this study.

## Author contributions

WP, BH, NG, YS, XY, and WW conducted the experiments. WP, YQ, HG, and JW analyzed the data and drafted the manuscript. JN, SD, LM, YQ, and LJ collected samples and epidemiology information. HG and JW conceived this study. All authors contributed to the article and approved the submitted version.
